# Living Skin Substitute Tissue—Is a Replacement for the Autograft Possible?

**DOI:** 10.3390/ebj4030031

**Published:** 2023-09-05

**Authors:** Angela L. F. Gibson

**Affiliations:** Department of Surgery, University of Wisconsin School of Medicine and Public Health, Madison, WI 53792, USA; gibson@surgery.wisc.edu

**Keywords:** skin substitute, allogeneic, autologous, cultured skin, burn, skin grafts

## Abstract

The ideal living tissue skin substitute for use in burn injury does not yet exist. The currently available alternatives to autologous skin grafting require an understanding of their characteristics and limitations to make an informed decision of surgical treatment options. In this review, living tissue substitutes are categorized by autologous and allogeneic cell sources and epidermal-only versus bilayered tissue options. A short summary of the tissue composition, clinical data, and indications is provided. Finally, the gap in technology is defined and future potential areas of research are explored.

## 1. Introduction

The search for a one-stage, off-the-shelf, readily available bilayered skin substitute for coverage of large burns has been ongoing for decades, and yet the ideal human skin replacement does not yet exist. Many attempts to create an ideal substitute have been reviewed extensively elsewhere [1,2,3,4]. Given no perfect option, it is imperative that we rethink what is necessary as an alternative to autologous skin grafting. Preservation of viable tissue during burn excision is a key tenet that will allow the use of the currently available options described below. Earlier determination of the need for surgical excision has the potential to reduce burn wound progression and thus preserve as much dermis as possible. Techniques that may allow replacement of autografts with the skin substitutes discussed below include the use of enzymatic debridement targeting only necrotic tissue and advanced diagnostic imaging to identify irreversible tissue necrosis while allowing the preservation and recovery of reversible cellular injury.

It should be noted that there is a fundamental problem with the skin substitute terminology in that it has become a catch-all phrase for any tissue, biologic treatment, or dressing that is used on acute and/or chronic wounds. This leads to confusion and inappropriate use of these products, leading to the assumption of failure when used in the wrong setting. For the purposes of this review, the focus will be on treatments that have the potential to replace the need for autografting in burn wounds. The products reviewed here are broken down by cellular content and by autologous versus allogeneic cell source, as organized in Table 1. 

## 2. Epidermal-Only Autologous Living Tissue

The cultured epithelial autograft (CEA) was the first autologous living tissue that became available for widespread use in patients with burns. The keratinocyte culture method was originally reported by Rheinwald and Green [5] in 1975, and the true life-saving clinical utility was initially realized in 1984 when the confluent sheets of keratinocyte cultures were used to save the lives of two young boys who suffered severe >97% total body surface area (TBSA) burns [6,7]. Later, access to this tissue was available through a company founded by Dr. Green starting in 1987 (BioSurface Technology, Inc., Cambridge, MA, USA) [8]. By the early 1990s, more than 240 patients had been treated with CEA and the technology was ultimately sold to Genzyme (Cambridge, MA, USA) in 1994 [9]. Reports of the “take” rate were variable; decreased rates were associated with infection and the long-term outcomes were anecdotally associated with friability. However, no large long-term studies are available. Of note, concerns about Marjolin ulcer have surfaced, with the literature suggesting an increased rate of malignant transformation of the CEA-grafted regions in half the time as the baseline squamous cell carcinoma transformation in severe burns [10,11].

The CEA was ultimately approved in 2007 under a Humanitarian Device Exemption (HDE) by the Centers for Device and Radiological Health and the Food and Drug Administration (FDA). The approval was later amended in 2016 to allow for profit sale by the Center for Biologics Evaluation and Research, with CEA “indicated for use in adult and pediatric patients who have deep dermal or full thickness burns comprising a total body surface area greater than or equal to 30%. It may be used in conjunction with split-thickness autografts, or alone in patients for whom split-thickness autografts may not be an option due to the severity and extent of their burns” [12]. Due to the HDE, the use of this product requires Institutional Review Board approval prior to each use in patients.

The culturing process begins when a patient is deemed a candidate, usually within 24–48 h of their injury. There are multiple CEA products approved across the world and the reader is directed to a recent review [13] as well as a website listing currently approved worldwide regenerative medicine products [14] to explore other specific product-related protocols. For the CEA (Epicel) supplied by Vericel (previously Genzyme), manufacturer instructions recommend obtaining a full thickness elliptical biopsy from a non-burned region to be shipped to the company for processing where the keratinocyte and fibroblast cell stocks are generated. Meanwhile, the patient’s burns are excised and temporary dermal replacements such as human cadaver allograft or acellular dermal matrices, such as Integra (Integra LifeSciences, Princeton, NJ, USA) or Biodegradable Temporizing Matrix (BTM) (Polynovo, Port Melbourne, VIC, Australia), are applied. A future operative date is chosen when the wound bed is deemed ready for grafting, and the tissues are grown over a course of 2–3 weeks to be available on the surgery date. If anything delays the surgery, a new date for culture preparation a few weeks later is chosen to allow a new batch of tissues to be generated. 

Histologically, these tissues continue to differentiate when fully healed after graft take and have multiple epidermal cell layers, including basal, spinous, granulosum, lucidum, and corneum. However, when they are initially grafted onto the patient after approximately 2–3 weeks in culture, the immature tissues (2–8 cell layers thick) lack a stratum granulosum and stratum corneum [12,15]. Keratinocyte cultures grown at the air/liquid interface have improved barrier function related to enhanced differentiation from the decreased humidity at the surface [16,17]. As experience has grown over the years, the initial advice to keep the wound bed moist is now traded for the goal to air out the CEA for long periods of time to encourage the maturation of the grafts. While there are no large-scale clinical trials, multiple case series have been published and with experience CEA can be a useful adjunct in very large burns without sufficient donor sites. However, it is very fragile and prone to breakdown. Since the initial approval, the postoperative care of CEA grafted tissues has evolved as burn surgeons have modified the initial protocols when the “take” of the epithelial tissue was suboptimal. 

Of note, the use of the CEA is not an effective option on burns that have been excised to fat or fascia without consideration for the dermal substrate that is necessary for the functional properties of skin. Prior to placement of the CEA, the wound must be prepared with an acellular dermal matrix (ADM) such as allograft with removal of epidermis [18], BTM [19] or Integra [20] prior to grafting of the CEA. This “in-vivo engineering” of what essentially amounts to a bilayered skin substitute likely contributes to the improved healing rates of the CEA compared to the early studies when the wound bed was not prepared with an ADM prior to placement of the CEA. 

Anecdotally, the use of CEA in conjunction with widely meshed autograft helps increase the overall healing rate and durability of the CEA sites [21]. Additionally, use of a dermal substitute to improve the wound bed is noted to enhance the take rate and improve overall functional outcomes [22]. While limitations such as fragility of skin during healing and blister formation are known, the CEA still remains a life-saving option for severely burned patients as was first identified in the early 1980s [6,7].

## 3. Bilayered Autologous Living Tissue

Replacing like with like is the goal with autograft coverage in burn injury; however, this requires creation of a donor site along with the resultant morbidity of an additional wound. Furthermore, in the case of large burns there may not be enough donor site available for complete coverage of the burn-injured tissue. An excellent recent systematic review describes in detail the various human trials using autologous bilayered skin substitutes and assesses their utility as epithelial stem cell niches [23]. Readers are referred there for more detailed information on culturing methods and clinical outcomes of these skin substitute trials. Currently, no bilayered autologous living tissues are approved for use; however, multiple trials of two different products have shown promise. The Self-Assembled Skin Substitute (SASS) is currently enrolling in a multicenter randomized clinical trial (clinicaltrials.gov NCT02350205) in Canada comparing SASS to autograft. From 2005–2014, SASS was available under a Special Access Program in Canada in a total of 14 patients with burns larger than 50% TBSA [24]. The tissue required two months’ preparation before grafting and entailed growing sheets of fibroblasts for 14–29 days, then combining three of these dermal-like tissues to provide a surface on which to culture keratinocytes for up to an additional three weeks. Finally, the tissue was lifted to the air–liquid interface for the final 9–14 days of culturing to enhance differentiation and barrier function. During this culture time, the wound bed was covered with allograft and replaced as needed. The tissues required ideal tissue culturing conditions without the ability for extended storage, such as refrigeration or cryopreservation, until placement onto the patient. The TBSA treated ranged from 420–6295 square centimeters and there was a mean SASS graft take of 98% with a reported 0% graft loss based on clinical observations [24]. Longer-term (up to 8 years) follow-up of this small cohort of patients did not reveal any challenges with functional restriction due to scarring, even in the children treated before puberty. A recent review authored by the trial’s principal investigator summarizes the history and current status of this tissue [25]. 

Another tissue in this category is an autologous engineered skin substitute (ESS) developed in the late 1990s [26]. Multiple studies by Dr. Boyce and team over the past 25 years have shown great promise of the ESS culturing method as a life-saving technology, albeit with similar culturing challenges noted above in SASS [27,28,29,30,31]. Despite these challenges, the bilayered tissue is superior to the CEA tissues likely due to the co-culture of epidermal and dermal layers [29]. The ESS tissue received Investigational Device Exemption (IDE), but initial attempts of studying this tissue were hampered by deficiencies noted by the FDA resulting in a Data Integrity Hold in 2007 that was subsequently lifted [31]. A study performed under this IDE from 2007–2010 evaluated 15 subjects who completed the study and found as early as postoperative day (POD) 7, the ESS closed the wounds with a functional epithelial barrier that was strong enough to tolerate therapy at POD 14 and the placement of pressure garments on POD 28 without loss of the ESS [31]. Overall, ESS engraftment in this study was noted to be 80%; while this remained significantly lower than autograft, the use of this technology was donor-site sparing. ESS received orphan drug designation by the FDA in June 2012; however, a Phase 2 clinical trial of this technology necessary for submission of a biologics license application to the FDA is listed as “not yet recruiting” despite the anticipated initiation of the trial in 2016 (ClinicalTrials.gov NCT01655407). 

In Europe, a recent Phase I clinical trial [32] has been completed using a bilayered skin tissue, referred to as EHSG-KF or denovoSkin (Cutiss, Schlieren, Switzerland), and Phase II clinical trials (Clinicaltrials.gov NCT03394612, NCT03229564, NCT03227146) are underway. In the initial safety trial, ten pediatric patients (one acute burn and nine reconstructive cases) were treated with a bilayered skin substitute consisting of autologous keratinocytes and fibroblasts cultured from a 4 cm^2^ split thickness skin graft harvested from the posterior auricular region. The tissues were grown by initial donor tissue disaggregation, isolation, culturing, and expansion of keratinocytes and fibroblasts. This was followed by creation of a dermis with bovine type 1 collagen hydrogels seeded with fibroblasts. The dermal equivalent underwent compression for a period of 5–6 days and then keratinocytes were seeded on the surface.The tissue was then allowed to mature for a period of 4–5 weeks. This safety trial grafted the skin substitute to small TBSAs (32–49 cm^2^) after preparation of the wound bed by either partial or full thickness excision. The grafts were assessed on POD 9-11, and complete wound healing was evaluated on POD 21. The authors noted 50–100% graft take at POD 21 for 8 patients, while 2 of the 10 study patients had 0% and 5% graft take related to complications resulting from patient non-compliance and hematoma, respectively. A promising unique feature of this substitute compared to the others reviewed here is that it incorporates a hydrogel base for the dermal component. However, the extent to how the addition of hydrogel to the dermal equivalent might improve outcomes has yet to be evaluated. 

Limitations of the bilayered living skin tissues include delay in definitive wound coverage due to the long preparation time; the patient often develops infection during this time, resulting in suboptimal graft take. The length of culture time required to generate fully differentiated tissue through cell expansion and tissue culture remains the significant obstacle, although additional studies continue to work towards reduced culture time without compromising the integrity of the tissue [25]. Also a concern that is not well defended in any of the studies is the need to harvest enough donor skin to be able to expand the cells with early passage primary keratinocytes without the theoretical risk of senescence given that later passage primary keratinocytes have decreased colony-forming efficiency [8]. Additionally, in all cultured tissue there is a lack of other cells and cellular structures known to be present in normal skin, an underdevelopment in the morphology of the dermal characteristics, such as rete ridges, and lack of distinction between papillary and reticular dermis. 

It should also be noted that these living cultures are very fragile. The details of the enhanced care required to graft the tissue and then subsequently enable engraftment on the patient are not clearly defined in any of the literature. While small, well-controlled cases appear to have great success, the ultimate implementation of these tissues in centers without the experience will likely have a steep learning curve, which risks inadvertent assumption of failure of the technology. It is clear that if any of these technologies make it to market, a bilayered living skin substitute will be the best option for treatment of full thickness burns. This is especially true in mortality reduction in patients with very large TBSA burns given that, unlike CEA or autologous cell suspension procedures (ReCell—Avita, Valencia, CA, USA), there is no widely meshed autograft underlay that is necessary. However, as with any complex technology, technical support for the early users will be necessary to enhance broad implementation of the tissues.

## 4. Bilayered Allogeneic Living Tissue

The benefit of allogeneic cells in the creation of a bilayered living tissue is the ability to generate large cell stocks at any time in advance of the need and allow “off-the-shelf” access that is impossible with autologous cells. There are two main tissues that have been approved for use in the United States for wounds and/or burns and are cultured with similar methods, although the exact culturing methodology is proprietary. The downside of allogeneic cells is that they do not represent a permanent replacement. However, they do act as a temporary biologic bandage to enhance wound healing through the regenerative mechanisms of their living cells. Therefore, their use is limited to wounds that contain intact dermal elements, as an overlay with meshed autograft, or in small wounds where closure occurs through re-epithelialization from the wound edges.

Apligraf (Organogenesis; Canton, MA, USA) is a bilayered living tissue sourced by normal neonatal human keratinocytes. It is classified as a device and approved for use in venous stasis ulcers and diabetic foot ulcers. The only published, non-case series clinical trial in burn injury studied 38 patients where Apligraf was used as an overlay treatment over widely meshed autograft compared to autograft with or without allograft overlay [33]. The authors concluded that use of Apligraf in this overlay manner improved pliability and Vancouver scar scores up to two years after treatment, suggesting that the tissue provided nutrients to decrease the scarring, although the exact mechanism is unknown. The study did not address the cost associated with the tissue and was not powered to look at patient differences. Of note, the original intention of the study was to use Apligraf alone without autograft; however, the Apligraf failure in the initial enrolled patients led to a revision of the protocol design. Ultimately, the need to continually source new cells is a downside of this technology, and without data to support efficacy in burn wounds, its use in burn wound healing was not further pursued.

The other commercially available allogeneic bilayered living tissue is StrataGraft (Mallinckrodt; Bedminster, NJ, USA), which is composed of allogeneic human neonatal foreskin cultured keratinocytes (NIKS—near-immortalized keratinocytes [34]) and dermal human neonatal foreskin fibroblasts in murine collagen. Multiple clinical trials (Phase 1–3) [35,36,37,38,39] have been published to provide data for FDA approval in deep partial thickness burns, with approval obtained in June 2021. The main findings of these trials support the fact that StrataGraft is donor site sparing in patients with deep partial thickness burns who would otherwise undergo autografting. Although the exact mechanism of action is unknown, it is likely a living tissue secreting growth-promoting substances for approximately a week, given that the initial Phase I/II clinical trial demonstrated that the tissue was viable at one week when it was removed from the patients [37]. However, at the three-month time point in subsequent clinical trial studies, a biopsy confirmed that the cells from the tissue were no longer present in the healed wound [39]. Multiple studies have demonstrated minimal side effects, most notably pruritis [38]. 

As with any technology, there are some challenges with extrapolating the data from the clinical trials to real-world use. First, the study populations were mostly composed of patients with small TBSA burns. It is unknown how this product would fare in a large TBSA burn, with the associated massive inflammatory response. A full thickness study was initiated in 2017 but was terminated early due to protracted enrollment and limited wound closure in the first three subjects (ClinicalTrials.gov NCT03005054). The current study that is recruiting for use of this tissue in full thickness burns is evaluating overlay of widely meshed autograft with StrataGraft (ClinicalTrials.gov NCT04765202) to determine the efficacy of this tissue in wounds without dermal elements. A pediatric Phase 3 study (ClinicalTrials.gov NCT05517902) evaluating the safety and efficacy of StrataGraft in pediatric patients who have deep partial-thickness burns is also currently enrolling patients.

An underexplored unique characteristic of the spontaneously immortalized near-diploid human keratinocyte cell line, NIKS, in StrataGraft tissue is the ability to stably genetically engineer the cells with beneficial genes for wound healing using non-viral methods to develop a therapeutic skin substitute [40,41]. Development of clonal populations of genetically stable NIKS cells through electroporation of host defense peptides such as Human Beta Defensin-3 and Cathelicidin, showed promise as anti-infective skin substitutes in animal models of wound healing [40,42]. 

Although unknown, one potential mechanism of action of StrataGraft on wounds is as a prolonged delivery system for growth factors. The ability to engineer this tissue to the individual needs of the wound would represent a significant breakthrough in treatments for acute and chronic wounds, including burns in complex patients with comorbidities that otherwise affect normal wound healing [41].

## 5. Gaps in Knowledge and Future Research

There is a plethora of products labeled as “skin substitutes” in the wound healing field. The challenge is matching the right product to the right patient. Factors including availability, cost, and wound bed preparation are essential to determining the best option of those that are clinically available. The current standard of visual inspection of the wound undoubtedly leads to errors in characterization of wound healing potential [43], and in doing so, may lead to unnecessary removal of viable tissue that would otherwise allow products such as CEA and StrataGraft to be more successful as stand-alone therapies. 

The bilayered autologous skin tissues (SASS, ESS and denovoSkin) represent the closest the field has come to the ideal skin substitute for burn injury. However, the challenges of the long complex culture methods and the lack of many of the structures in the skin are not negligible. With the rapid advances in porcine xenotransplantation technologies, the potential for use of a porcine source of tissue for permanent skin transplantation is very real [44,45]. Early-stage investigation of gene-edited porcine xenotransplantation, currently ongoing, may represent a sustainable option as an easily sourced ideal living skin substitute with the potential for long-term engraftment. A Phase I/II open-label, multicenter clinical trial to evaluate the safety, tolerability, and efficacy of Xeno-Skin^®^ as a temporary wound closure in 15 patients with severe burns is listed as having completed recruitment on ClinicalTrials.gov (NCT03695939), but no results are currently posted. Continued advancement in the field of living skin substitutes must engage a multi-disciplinary team of burn surgeons and scientists using a translational research approach to understanding the tissue microenvironment in burn wounds. This will better allow for the development and uptake of products that have the potential to reduce suffering and save lives in severe burn injury.

## Figures and Tables

**Table 1:** Living skin substitutes.

	Pros	Cons	Commercially Available Approval Status for Burn Indication
Epidermal autologous living tissue	Shorter time to culture (2–3 weeks) Can be used in combination with widely meshed autograft to improve wound closure rates	Fragile Requires dermal substitute base preparation which can increase time to grafting, but may increase ultimate graft take Expensive	Epicel (United States) approved for deep dermal or full thickness burns ≥ 30% TBSA JACE (Japan) approved for deep dermal or full thickness burns ≥ 30% TBSA Keraheal (Republic of Korea) approved for 2nd degree burns
Bilayered autologous living tissue	Better durability than epidermal only Long-term incorporation	Longer time to culture (4–6 weeks) Not available off the shelf Expensive	SASS (Canada) not currently approved ESS (United States) not currently approved denovoSkin (Europe) not currently approved
Bilayered allogeneic living tissue	Better durability than epidermal only Readily available stock on demand May enhance healing due to active release of growth factors	Not incorporated in wound long term Requires dermal elements for healing in burns Expensive	Apligraf (United States) not currently approved for burns StrataGraft (United States) approved for deep partial-thickness burns with intact dermal elements

## Data Availability

Not applicable.
